# Patient involvement in diabetes care: experiences in nine diabetes care groups

**DOI:** 10.5334/ijic.2207

**Published:** 2015-12-15

**Authors:** Lidwien C. Lemmens, Simone R. de Bruin, Jeroen N. Struijs, Mieke Rijken, Giel Nijpels, Caroline A. Baan

**Affiliations:** National Institute for Public Health and the Environment, Centre for Nutrition, Prevention and Health Services, Bilthoven, The Netherlands; National Institute for Public Health and the Environment, Centre for Nutrition, Prevention and Health Services, Bilthoven, The Netherlands; National Institute for Public Health and the Environment, Centre for Nutrition, Prevention and Health Services, Bilthoven, The Netherlands; NIVEL Netherlands Institute for Health Services Research, Utrecht, The Netherlands; Department of General Practice, EMGO Institute VU University Medical Centre, Amsterdam, The Netherlands; National Institute for Public Health and the Environment, Centre for Nutrition, Prevention and Health Services, Bilthoven, The Netherlands; Scientific Centre for Transformation in Care and Welfare (Tranzo), University of Tilburg, Tilburg, The Netherlands

**Keywords:** patient involvement, diabetes, care groups, bundled payment, integrated care

## Abstract

**Introduction:**

Despite the expected beneficial effects on quality of care, patient involvement in diabetes care groups, which deliver a bundled paid integrated care programme for diabetes type 2, seems to be limited. The aim of this study was to gain insight into levels and methods of patient involvement, into facilitators and barriers, and into the future preferences of care groups and patient representatives.

**Theory and methods:**

Semi-structured interviews were held with 10 representatives of care groups and 11 representatives of patient advocacy groups. An adapted version of Arnstein's ladder of citizen participation was used to define five levels of patient involvement.

**Results:**

Patient involvement in care groups was mostly limited to informing and consulting patients. Higher levels, i.e., advising, co-producing and decision-making, were less frequently observed. Care groups and patient representatives perceived largely the same barriers and facilitators and had similar preferences regarding future themes and design of patient involvement.

**Conclusion:**

Constructive collaboration between diabetes care groups and patient representatives to enhance patient involvement in the future seems viable. Several issues such as the lack of evidence for effectiveness of patient involvement, differences in viewpoints on the role and responsibilities of care groups and perceived barriers need to be addressed.

## Introduction

Patient-centred care is an important paradigm in current health care systems [1]. In Western countries, patients are therefore increasingly encouraged to contribute to the planning, development and delivery of health care [2–5]. Underlying these developments is the belief that involving patients leads to increased accessibility and quality of care and to improved health and quality of life [4,6].

Patients can be involved in health care in different ways and on different levels. Several concepts such as patient engagement, patient activation, shared decision-making, patient involvement and patient empowerment are associated with the role of patients in health care. Definitions of these concepts may vary between settings and studies [7–9]. In the current study, we defined *patient engagement* as shared decision-making by patients and health care professionals on an *individual level* about patients’ health, their personal goals and priorities, and their individual care process [7,10,11]. *Patient involvement* was defined as the involvement of patients in decision-making on a *collective level* on various topics such as policy-making, research agenda setting and planning and delivery of health services [4,6,12]. The focus of the current study is on patient involvement, and more specifically on patient involvement in care groups.

Care groups are new actors in the Dutch health care system; they emerged after the introduction of a bundled payment approach for integrated chronic care [13–15]. A care group is a legal entity owned by multiple health care providers who are mostly general practitioners. Care groups offer care programmes for one or more chronic illnesses such as diabetes and Chronic Obstructive Pulmonary Disease (COPD). The care group either delivers the care programme itself or subcontracts other health care providers. The care programme for diabetes type 2 was the first to be enrolled. This programme is based on the Dutch Diabetes Federation Health Care Standard, which defines requirements for standard diabetes care and is approved by all national provider and patient associations [13,16]. Services offered within the care programme are standard periodic check-ups (three-monthly check-ups and full annual check-up), yearly eye and foot examinations, dietary counselling, additional diabetes-related GP consultations and certain advisory services by internal medicine specialists (internists) [17]. Currently, there are about 100 care groups with a care programme for diabetes in the Netherlands [18]. A care group is comparable to Accountable Care Organizations in the USA or Clinical Commissioning Groups in the UK [13,15,19].

In integrated care, a central role for patients is important, in addition to good coordination and cooperation between health care providers [16,20,21]. Enhancing patient involvement aims to give patients more influence on the content and organisation of the care process. The expectation is that this results in more demand-driven and thereby more person-centred care, which can ultimately improve quality of care and patient outcomes [4,6,22]. Recent studies among diabetes care groups reveal that care groups are still seeking ways to involve patients within their organisation [14,17,23,24]. To date it is unknown why patient involvement is still limited and what factors facilitate or impede patient involvement. It is further unknown whether care groups want to attribute an active role to patients and how they should shape this role. Moreover, it is unknown whether patients themselves actually want to be involved in such processes and if so, to what degree and in what ways they wish to participate. These insights are becoming even more important as care groups are increasingly implementing care programmes for various other chronic diseases [14,17,23].

The aim of our study was to gain insight into: (i) the levels and methods of patient involvement in diabetes care groups, (ii) the facilitators and barriers for patient involvement and (iii) the preferences of care groups and patients with regard to future patient involvement.

## Methods

### Study setting

The present study was performed within nine diabetes care groups in the Netherlands that were part of the evaluation study of the bundled payment approach for diabetes type 2 before it was implemented nationwide in 2010 [14,17]. These care groups were then selected based on their size, geographical location and organisational structure and were considered a representative sample of Dutch care groups.

### Study design and population

In this qualitative study, semi-structured interviews were conducted with representatives of the nine care groups and regional patient advocacy groups. The representatives of the care groups had to be the persons who were responsible for patient involvement within their organisation. During the interviews, these representatives were asked for their contact persons of the regional patient advocacy groups. These patient representatives where then invited for the interviews as well. All interviews were conducted between October and December 2011.

### Theoretical framework

This study had an explorative character and follows a general inductive approach with regard to the experiences and preferences related to patient involvement [25]. For the levels of patient involvement, the “participation ladder” was used as a framework. This ladder is adapted from Arnstein's ladder of citizen participation to make it suitable for application in a health care setting [26–28]. It was assumed that care groups have a similar management layer as other types of health care organisations that is responsible for decision-making regarding health policy, quality management and delivery of care. Therefore, it was assumed that similar levels of patient involvement would apply. The ladder consists of five steps that represent five steps of patient involvement defined as levels of decision power (Table 1). These levels range from passive participation (step one: being informed about decisions) to very active participation (step five: participating in formal decision-making). The definition of the levels and the patients’ role are described in Table 1. Although the term “ladder” implies so, it is not necessary to go through the levels in succession or to go through all preceding steps to reach the highest level.

### Interviews

Interview item lists were constructed based on literature and on recent studies in care groups [17,23,28,29]. The aim of the interviews was to collect information about experiences and preferences of both care group and patient representatives regarding patient involvement. The item lists included the following items, which were elaborated in open questions (see Appendices):

1. *Current situation*: (i) the extent to which patients are informed about or involved in decision-making on a collective level in the care group on various topics such as the planning and delivery of the care programme or the care purchasing process and (ii) the nature and extent of the current interactions between patient representatives and care groups;
2. *Facilitators and barriers:* (i) types of perceived facilitators and barriers regarding patient involvement in the current situation;
3. *Future preferences:* (i) preferences regarding patient involvement in care groups in the future.

As we wanted the respondents to speak about patient involvement without restraint, we did not inform them about what is considered the “right” level of patient involvement according to the Governance Code or specific laws for health care institutions (i.e., step three: advising by means of a client council) [14]. Each interview was conducted by two researchers (L.L. and S.dB.) trained in qualitative research and was audio recorded. A summary of each interview was alternately made by one researcher and checked by the other. These summaries were sent for approval to the interviewees. If necessary, the summaries were adapted.

### Data analysis

Two researchers (L.L. and S.dB.) independently assigned the levels of patient involvement to each summary by hand using the participation ladder. In addition they assigned overarching themes to the experienced barriers and facilitators and the preferences for future patient involvement. These assigned levels of patient involvement and themes related to factors and preferences were then compared, and consensus was reached when necessary.

General care group characteristics i.e., the number of diabetes patients enrolled in the care programme and the period that the care programme was offered were compared to interpret differences between care groups with regard to the level of patient involvement. Experiences and preferences of representatives of care groups and patients were compared to gain insight into the extent to which their ideas corresponded. This comparison provided insight in the steps needed to realise more active levels of patient involvement within care groups.

## Results

### Respondents

Ten representatives of nine care groups and 11 representatives of nine patient advocacy groups were interviewed (*n* = 21). Representatives of the care groups included general managers (*n* = 6), a programme leader (*n* = 2), a programme assistant (*n* = 1) and a quality/process manager (*n* = 1). Patient representatives included board members of regional patient associations (*n* = 5), (policy) advisors of regional umbrella organisations of patient associations (*n* = 3), members of a client council (*n* = 2) and a chairman of a supervisory board of a care group who was also vice president of a patient association (*n* = 1).

### Characteristics of care groups

The period that the care groups were offering the care programme ranged from two to seven years (Table 2). The number of GPs participating in the care groups varied from 35 to 130. The number of diabetes patients enrolled in the care programme varied from 2400 to 15,250.

### Current situation: levels and methods of patient involvement

The level of patient involvement differed between the care groups. In six of the nine care groups, patients participated at more than one level (Table 2). These care groups each showed combinations of different levels of patient involvement. Overall, care groups with smaller patient numbers did not differ from care groups with higher patient numbers with regard to the level of patient involvement. The three care groups that offered the diabetes care programme for a longer period (since 2005 or 2006) showed high levels of patient involvement (step four or five), and the two care groups that offered the programme for a shorter period (since 2009) showed only low levels (step one or two). However, there were also two care groups that offered the programme for a relatively long period (since 2006 or 2007) but in which patient involvement was still limited to the lowest level (step one).

Representatives of all care groups stated that patients were informed about the care group, the participating health care providers, and the services to which the patients were entitled within the care programme (step one). In all care groups, the information was provided on a structural basis through the care groups’ website and through brochures or information letters that were provided to patients when they enrolled in the care programme. One care group additionally organised yearly information meetings for their patients. In three of the nine care groups, involvement of patients was limited to this lowest level.

Representatives of five care groups stated that the care groups also consulted patients (step two). In four of these care groups patients were consulted by patient surveys (e.g., CQ-index). Other methods to consult patients were meetings with patient associations (*n* = 3), patient panels (*n* = 2) and reflective conversations (*n* = 1). Patients were mostly consulted ad hoc in specific project, for example to gain insight into patient experiences with an electronic patient record.

Representatives of three care groups reported to ask patients for their advice (step three). In two of these care groups, a client council was structurally embedded within the organisation. In one of these two care groups, the client council was asked for its advice regarding current issues within the care group such as their reorganisation and its consequences for patients. The client council of the other care group mainly acted as a sounding board for new ideas, the development of information brochures, and initiatives to improve diabetes care. The third care group occasionally asked the client council of an affiliated hospital for advice. This client council was asked to advice on specific issues such as the role of medical specialists in the diabetes care programme.

Representatives of two care groups mentioned that co-production took place with the national association for diabetes patients (step four). In one care group, this concerned a once-only project to improve the diabetes care process for patients. In the other care group, this concerned structural co-production in ongoing conjoint projects and initiatives including the development of brochures, development of a course for diabetes patients and a project to gain insight into preferred glucose meters.

A representative of one care group mentioned formal decision-making with patients about organisational matters (step five). In this care group, a patient representative was the chair of the supervisory board of the care group. This board consisted of four members in total, the others being two general practitioners (GP’s) and one boardmember of an affiliated hospital. The patient representative had a final vote in the policy-making of the care group. However, he was not a patient of this specific care group but of another care group.

The board members of the regional patient associations as well as the (policy) advisors of regional umbrella organisations of patient associations stated that they participated in several care groups in their region. It differed per region whether this involvement was initiated by the patient representatives or by the care group. The involvement of the patient representatives in the care groups could have either an incidental or structural character. In some regions, the care group, the regional patient association and the health insurer signed a covenant to collaborate regularly on themes including organising self-management support and using patient feedback.

### Facilitators and barriers for patient involvement

Perceived facilitators and barriers corresponded largely between representatives of care groups and patients (Table 3). In general, facilitating and limiting factors regarded the themes motivation, competences, the choice of the method of patient involvement or resources.

#### Motivation

Both representatives of care groups and patients indicated that sufficient motivation of the parties involved is important. It was assumed by most representatives of both parties that patient involvement could be an instrument to improve quality of care. Therefore, most of them were committed to shape patient involvement. However, a representative of one care group experienced that it was already difficult to motivate patients to be engaged at an individual decision-making level, let alone on a collective decision-making level. Representatives of other care groups stated that being actively approached by patient representatives with realistic ideas about patient involvement would be facilitating. In contrast, some patient representatives shared the opinion that care groups were not open to their ideas about (innovative) patient projects or that they lacked entrepreneurship. Moreover, some representatives of care groups stated that patient involvement had lower priority than other organisational matters or than patient engagement in the individual care process.

#### Competences

Representatives of both care groups and patients stated that specific competences of patient representatives are important for successful patient involvement. Patients should be able to look beyond their individual care process and to promote the collective interests of a patient group. Furthermore, they should be capable to act as an equal conversation partner for care groups, to be taken seriously. Representatives of patient associations stated that they had a special programme to educate patient representatives about the organisational structure of care groups and about the bundled payment approach. Both care groups and patient representatives mentioned that they experienced difficulties finding patients with the right competences.

#### Methods

How patient involvement is shaped was also important according to representatives of care groups and patients. Both parties indicated to prefer collaboration within concrete projects. Moreover, the themes of these projects should be useful and relevant to both care groups and patients. Representatives of patients further indicated that a long-term vision of care groups on patient involvement would be helpful for embedding patient involvement in the organisation.

#### Resources

According to both representatives of care groups and patients, resources for patient involvement are limited. Representatives of care groups mentioned that they had trouble to obtain budget for patient involvement. Moreover, patient representatives stated that they experienced cutbacks on government funding of patient associations. Some representatives of care groups further mentioned that some patient associations did not always have a contact person in their region available. Patient representatives on their turn stated that care groups did not always have sufficient personnel to be engaged in patient involvement.

### Future preferences for patient involvement

Representatives of care groups and patients had rather similar preferences with regard to the future design of patient involvement. Both parties want to collaborate in conjoint projects around self-management support to enable diabetes patients to manage their chronic illness better. Both representatives of care groups and patients further indicated wanting to establish more structural contact with each other. They aimed at a combination of regular meetings, for example biannual consultations, and cooperation in occasional projects around specific themes such as supporting self-management or building a patient portal. Care groups as well as representatives of patients also mentioned wanting to collaborate with other patient groups or associations. Such collaboration was considered of particular importance since care groups are increasingly implementing care programmes for other chronic diseases in addition to diabetes, such as COPD and vascular risk management. Finally, both representatives of care groups and patients liked to explore whether social media could be used to collect patient experiences and feedback or to recruit patients for becoming a patient representative in a client council or patient association.

## Discussion

### Discussion of the results

We aimed to gain insight into patient involvement in diabetes care groups. Our study showed that patient involvement in care groups mostly concerned informing patients, which is the lowest and most passive level of involvement, and consulting patients, which is the first level of more active involvement [26,27]. Higher levels, i.e., patients giving advice, co-producing or decision-making, occurred less frequently. Results of international studies in other types of health care organisations also show that patients are mostly only consulted [3,7].

The nine care groups showed a variety of (combinations of) levels for patient involvement. Several reasons may explain this observed variety. A first explanation is the variety in the period that care groups were offering the diabetes care programme. Care groups that offered the programme for a longer period more often showed a higher level of patient involvement than care groups that offered the programme for a shorter period. It seems that these care groups have their organisational structure and care programme(s) better on track and therefore are able to engage themselves in patient involvement and prioritise it.

A second explanation for the observed variety in levels is the difference in opinion of care groups about their role and responsibilities regarding patient involvement. This difference in opinion seems, at least partially, to result into different levels of patient involvement in care groups. Some care groups stated to only inform their patients. These care groups thought that they should not bother patients with decision-making on a collective level or they thought that the GP practices that are member of the care group are responsible for patient involvement. Other care groups thought they should involve patients as much as possible into their organisation. These care groups showed higher and more active levels of patient involvement and for example had a client council, did conjoint projects with patient associations or had a patient in the board of trustees.

The facilitators and barriers for patient involvement as experienced by representatives of care groups and of patients largely corresponded. The experienced facilitators and barriers regarded the themes motivation, competences, resources and characteristics of the patient involvement method and are in line with those described in international literature on patient involvement in other types of organisations, such as health care centres and mental health services [3,7,11,30,31]. This implies that these may be universal for patient involvement in health care and are not confined to integrated care organizations such as care groups. Care groups, but also similar organisations such as Accountable Care Organizations in the US and Clinical Commissioning Groups in the UK, might learn from other types of longer existing organisations when it comes to resolving barriers for patient involvement. For example, they could ask an affiliated hospital how they have recruited patients for their client council and what competences are deemed the most important for these patient representatives.

Both representatives of care groups and of patients appear to be committed to further design patient involvement. Both parties assume that patient involvement could be an instrument to improve quality of care. The preferences of care groups and patient representatives are largely similar regarding the design of patient involvement in the future. Common goals include realisation of concrete projects around organising self-management support, structural consultation of patient representatives, involvement of various patient groups and experimenting with social media. These common goals seem to form a solid basis for constructive collaboration between care groups and patient representatives in the future. Moreover, representatives of patient associations could guide care groups in shaping patient involvement as these representatives often already have some experience with patient involvement in other care groups or health care organisations. Furthermore, care groups could prioritise patient involvement within their organisation and allocate part of the bundled payment budget to it, as they are free to spend this budget as they find appropriate. They could spend this money for example on educating regional patient representatives about the organisational structure of care groups or on setting up a patient panel. This seems especially important, as patient associations have very limited financial resources themselves due to cutbacks.

However, to be able to further shape patient involvement some issues need to be addressed. First, the formulation of an unambiguous point of view about the role and responsibilities of both care groups and patients in decision-making processes on a collective organisational level is needed, as this study showed that different viewpoints cause a variety of levels of patient involvement. Second, although both care groups and patient representatives assume that patient involvement could be an instrument to improve quality of care, this is not supported by evidence. Due to the limited amount of research on care groups, it is still largely unknown what patient involvement on a collective level in care groups can bring in terms of effects on the quality of care. In addition, studies investigating patient involvement in other types of care organisations show a lack of evidence for the effectiveness of patient involvement [4,28]. More research about the benefits of and preconditions for active patient involvement is needed. As is research to identify what levels and methods of patient involvement sort the best results with regard to the improvement of quality of care. In addition, it should be studied what competences of patient representatives are needed to be successfully involved in decision-making on a collective level on one hand and still being representative for all patients on the other hand [2,6]. Moreover, not only clinical outcomes should be measured but also patient relevant outcomes such as social participation or experienced autonomy [32].

### Strengths and limitations

In this study the “participation ladder” was used to assign the levels of patient involvement. There has been some criticism on this ladder with regard to its sole focus on the extent of decision power citizens or patients have, thereby not acknowledging the value of the process and the diversity of knowledge and experience of both health care providers and patients [33]. However, we have tried to overcome this by using the participation ladder as a pragmatic starting point and additionally using a general inductive approach with regard to experiences and preferences concerning patient involvement in care groups.

In other studies, only representatives of care groups were questioned about patient involvement [14,17,24]. By interviewing representatives of care groups as well as patient representatives, both perspectives on patient involvement are represented. By comparing ideas about patient involvement, perceived facilitators, barriers and future preferences insight was gained into the extent to which views corresponded between both parties. This proved to be very useful, as results show that these views are quite similar and could form the basis of a constructive collaboration in the near future.

In this study, representatives of 9 out of 100 care groups were interviewed, which is only a small sample. However, these nine care groups were not chosen randomly but were selected because they all participated in an evaluation study, in which these care groups were regarded as representative sample based on their size, geographical location and organisational structure [14,17]. Nevertheless, a recent study under all Dutch care groups revealed that these nine care groups show on average higher levels of patient involvement than other care groups [23]. Consequently, the results of this current study cannot be regarded as representative for all care groups. Still, the experiences of the nine care groups can provide useful information for other care groups and for similar organisations in other countries as Accountable Care Organizations and Clinical Commissioning Groups.

## Conclusion

Patient involvement in diabetes care groups is mostly limited to informing and consulting of patients. Both care groups and patient representatives assume that patient involvement is an instrument to improve quality of care and are therefore committed to collaborate with each other. Moreover, they have similar preferences regarding future themes for and shaping of patient involvement. This can offer a basis for a constructive collaboration between both parties, especially when care groups prioritise patient involvement and make use of the experiences of patient associations with patient involvement. However, several issues such as the lack of evidence for effectiveness, differences in viewpoints on the role and responsibilities of care groups and perceived barriers for patient involvement need to be addressed.

## Figures and Tables

**Table 1.**
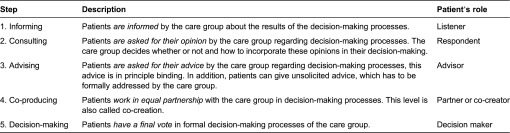
Steps of the participation ladder

**Table 2.**
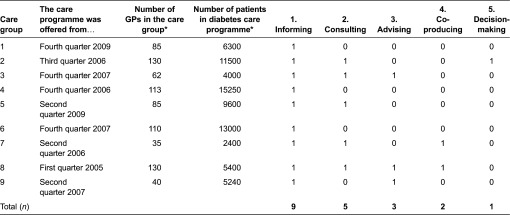
Care group characteristics and their levels of patient involvement

0 = no and 1 = yes

*As reported in fall 2011

**Table 3.**
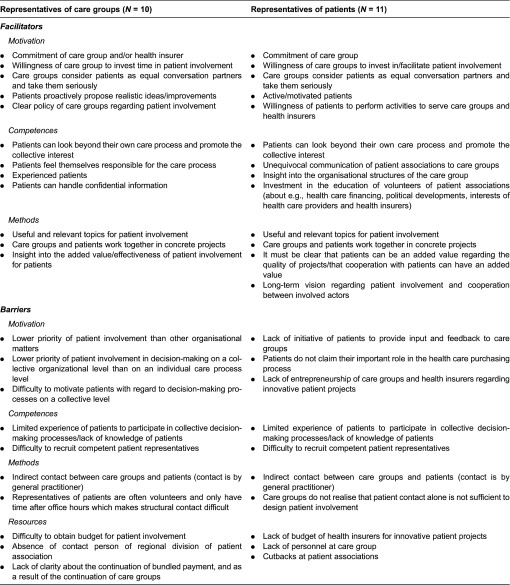
Perceived facilitators and barriers for patient involvement

## Appendices

### Item list for interview care groups

#### Current situation

1) To what extent are there interactions between your care group and patient representatives? What themes or issues are being discussed during these interactions (e.g., quality of diabetes care or (re-)design of the diabetes integrated care programme)?

2) Are patients informed about or involved in decision-making processes about the organisation of diabetes care within your care group? If yes, how are patients being informed or involved (e.g., information evening, participation evening, advisory board, client council or supervisory board)?

3) To what aim are patients involved in decision-making and in what kind of decision-making processes are patients being involved (e.g., decisions about the content of the diabetes disease management programme, contracting of preferred providers, health care purchasing and agreements on task substitution of care professionals)?

4) How often are there interactions between your care group and patient representatives or are patient involved in decision-making processes?

5) What role does the regional patient association play?

#### Pros and cons and satisfaction

6) What are the pros and cons of the way you are currently interacting with the patient representatives and of the way patients are currently being involved in decision-making processes?

7) Are you satisfied with the way you are currently interacting with patient representatives and with the way patients are currently being involved in decision-making? Could you explain why or why not?

#### Future wishes

8) Do you intend to shape your interactions with patient representatives in a different way in the future? Or do you intend to involve patient representatives in a different way in decision-making processes? Could you explain why or why not? And in what way?

#### Barriers, facilitators and preconditions for patient involvement

9) What do you see as barriers and facilitators of patient involvement in care groups?

10) What preconditions have to be met to realise patient involvement in care groups?

#### Recommendations for implementing patient involvement

11) What would you like to recommend, based on your own experience, to other care groups?

### Item list for interview with patient representatives

#### Current situation

1) To what extent are there interactions between patient representatives and care groups? What themes or issues are being discussed during these interactions (e.g., quality of diabetes care or (re-)design of the diabetes integrated care programme)?

2) Are patients informed about or involved in decision-making processes about the organisation of diabetes care within the care group? If yes, how are patients being informed or involved (e.g., information evening, participation evening, advisory board, client council or supervisory board)?

3) In what kind of decision-making processes are patients being involved (e.g., decisions about the content of the diabetes disease management programme, contracting of preferred providers, health care purchasing and agreements on task substitution of care professionals)?

4) How have interactions between patient representatives and care groups been established and how have patient representatives been involved in decision-making processes of care groups?

5) For what reasons did you want to be involved?

6) Are there any interactions or collaborations with other patient representatives?

#### Pros and cons and satisfaction

7) What are the pros and cons of the way you are currently interacting with the care groups and of the way patients are currently being involved in decision-making processes?

8) Are you satisfied with the way you are currently interacting with care groups and with the way patients are currently being involved in decision-making? Could you explain why or why not?

#### Future wishes

9) In the future, would you like to be involved in a different way in decision-making processes within care groups than you are now? Could you explain why or why not? And in what way?

#### Barriers, facilitators and preconditions for patient involvement

10) What do you see as barriers and facilitators of patient involvement in care groups?

11) What preconditions have to be met to realise patient involvement in care groups?

#### Recommendations for implementing patient involvement

12) What would you like to recommend, based on your own experience, to other patient representatives?
